# Minimal access surgery in children – 5 years institutional experience

**DOI:** 10.4103/0972-9941.18996

**Published:** 2005-09

**Authors:** S N Oak, S V Parelkar, T Akhtar, M Joshi, R Pathak, N Viswanath, K Satish Kumar V, K Ravikiran, L Manjunath, A Ahmed

**Affiliations:** Department of Paediatric Surgery, T.N.M. C and B.Y.L. Nair Hospital, Mumbai, India

**Keywords:** laparoscopy, minimal access surgery, video assisted thoracoscopic surgery

## Abstract

**Context:**

Minimal access surgery (MAS) in children are common place and performed worldwide with gratifying results as the learning curve of the surgeon attains plateau. We share our experience of this technically evolving modality of surgery, performed at our setup over a period of 5 years. We also review and individually compare the data for commonly performed procedures with other available series. Author also briefly discuss potential advantages of MAS in certain debatable conditions performed quickly and with cosmesis as open procedure.

**Materials and methods:**

We performed 677 MAS in children aged between 7 days and 12 years. Five hundred and sixty-eight of these were Laparoscopic procedures and 109 were Video assisted thoracoscopic surgeries (VATS). In all laparoscopic procedures, the primary port placement was by the Hasson's open technique. We have used 5, 3 and 2 mm instruments. Our study include 259 inguinal hernia, 161 Appendectomies, 95 VATS for empyema, 51 orchiopexies, 49 diagnostic laparoscopy, 29 cholecystectomies, 22 adhesionlysis and other uncommonly performed procedures.

**Results:**

The ultimate outcome of all the performed procedures showed gratifying trend, the data of which are discussed in detail in the article. Conclusion: As we gained experience the operating time showed a decreasing trend, the complication rates and conversion rate also reduced. The advantages we came across were better postoperative appearances, less pain and early return to unrestricted activities.

The role of minimal access surgery (MAS) in surgery of children is slowly emerging over the last decade. The scope of MAS is to minimize the traumatic insult to the patient without compromise of the safety and efficacy of the treatment. For over two decades, paediatric MAS was restricted mainly to diagnostic use. More recently, however, with increasing experience and advances in miniaturized instrumentation, the role of MAS in the modern paediatric surgical armamentarium has become accepted. The paediatric surgical community has embraced minimal access techniques for some operations; others remain controversial. With each advancing year, the instruments and optics are being refined and newer indications are getting added to the list of laparoscopically manageable maladies.

## MATERIALS AND METHODS

Six hundred and seventy-seven children aged between 7 days and 12 years underwent MAS in Paediatric Surgery Department of a University teaching Hospital between June 2000 and March 2005. Five hundred and sixty-eight of these were laparoscopic procedures and 109 were Video assisted thoracoscopic surgery (VATS) [Table 1].

**Table 1:** Showing spectrum of cases

Surgery	Number
Inguinal hernia repair (number of repairs)	259
Appendectomy	161
VATS for empyema	95
Orchiopexy	51
Diagnostic laparoscopy	49
Cholecystectomy	29
Adhesions	22
Pullthrough for hirschsprungs	13
Ovarian tumours and cysts	10
Mesenteric lymph node biopsy	8
Nephrectomy	6
Pullthrough for ARM	6
Fundoplication	5
Splenectomy	5
VATS lung biopsy	5
Congenital diaphragmatic hernia	4
Liver abscess	4
Lung hydatid	4
Meckel's diverticulum	4
VATS aspiration of abscess	4
Mediastinal mass	2
Mesenteric cyst	2
Pancreatic pseudocyst	2
Primary peritonitis	2
Pyeloplasty	2
VP Shunt with pseudocyst	2
Adrenelectomy	1
Cholangiogram	1
Faecal fistula	1
Hydrometrocolpos	1
Intersex-gonadectomy	1
Intussusception	1
Ovarian torsion	1
Parovarian cyst	1
PD Catheter	1
Pyloromyotomy	1
Retroperitoneal tumour biopsy	1
Ureterocoele	1
Varicocoele	1
VATS in Chest trauma (bronchial tear)	1

In all laparoscopic procedures, the primary port placement was by the Hasson's open technique. The abdomen was insufflated with prewarmed carbon dioxide starting at a pressure of 6 mmHg and slowly increased to 12 mmHg depending upon the age and weight of the child. A 5 mm wide-angle 0° telescope was used. We have used 5, 3 and 2 mm instruments. Placement and number of secondary ports were dependent on the area of interest taking utmost care in triangularization. A thorough diagnostic laparoscopy was done once the scope was inserted including the walking of the bowel to rule out any bowel pathology. Then the procedure was completed depending on the pathology.

Hundred and nine children underwent VATS. The scope was inserted through the tube thoracostomy site in cases where it was inserted preoperatively but in the other patients it was inserted in the mid-axillary line at the sixth or seventh intercostal space. In empyema, most of the time for toileting and even for decortication we have used only two ports.

## RESULTS

### Laparoscopic inguinal herniorrhaphy

This series includes 200 children with inguinal hernia treated laparoscopically (158 males, 41 females and 1 intersex). The age range was from 1 month to 12 years. Total of 259 hernias were repaired [201 in male, 56 in female, 2 in a child with intersex (CAIS)]. Twenty-one of the patients who presented with left hernia and 32 of those who presented with right hernia (totally 53) had a contralateral patent processus vaginalis (PPV). In three patients, both the internal rings were closed. In female, majority were repaired using an endoloop and the redundant sac excised. But in three female hernias suturing with extracorporeal knotting was done. The type of repair in males has progressed from just dissection to intracorporeal suturing (darning or purse-string) with inracorporeal knotting to intracorporeal suturing with extracorporeal knotting without dissection. Initially we used to use absorbable vicryl and in two of these cases we had recurrence. Now we use 3-0 Ethilon^®^ nonabsorbable sutures. We have repaired 62 hernias with extracorporeal knotting using 3-0 Ethilon in which we did not have any recurrence. The operating time showed a decreasing trend as the team gained experience. A unilateral repair required 25 min and a bilateral repair 35 min. There were four conversions to open herniotomy in our study. In three cases we felt the repair was not secure and in one case there was bleeding from inferior epigastric vessel. We had nine recurrences mainly in the earlier phase of study while using the intracorporeal suturing with intracorporeal knotting with absorbable suture technique during the learning curve giving a total recurrence rate of 3.5%. After shifting to extracorporeal knotting and avoidance of absorbable sutures, there are no recurrences. Four patients had postoperative hydroceles which resolved spontaneously within 2 months. One patient with persistent hydrocele after 3 months underwent open herniotomy. Doppler at 3 months after surgery showed no testicular atrophy.

### Laparoscopic appendicectomy

**Figure 1:**
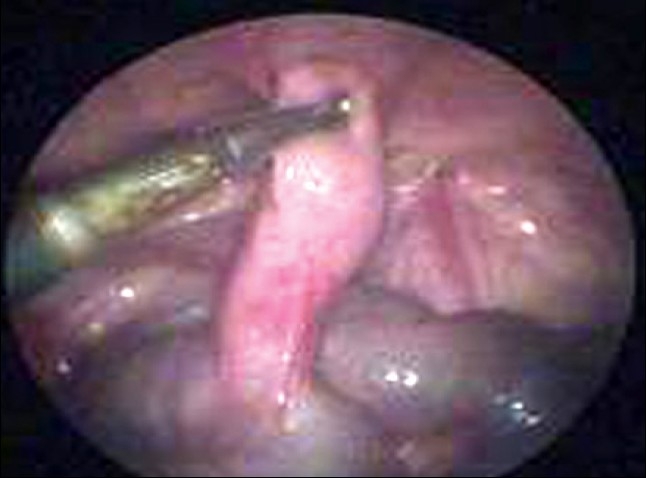
Meckel's diverticulum

**Figure 2:**
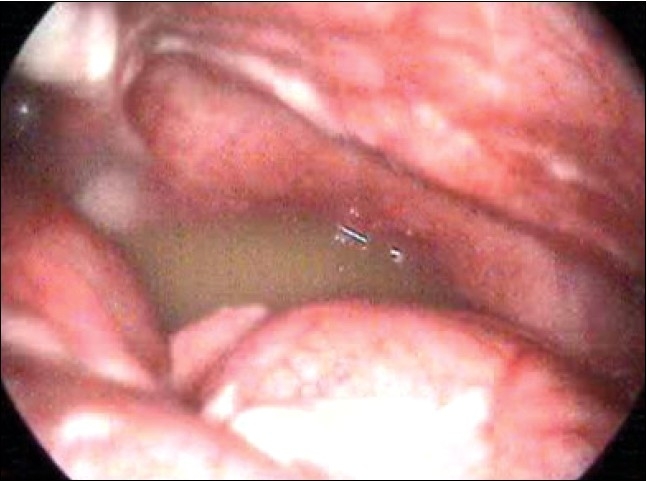
Frank pus in pelvis in a case of primary peritonitis

Two hundred and ten children presented with history of abdominal pain. Of these 161 children had features of acute appendicitis for whom laparoscopic appendicectomy. The remaining 49 children who presented with Recurrent abdominal pain underwent diagnostic laparoscopy which revealed features of Appendicitis in 11 cases, adhesions in 22 cases, Kochs abdomen in five cases, Twisted ovarian cyst in three cases, Meckels in two cases [Figure 1], pelvic peritonitis in one case [Figure 2] and in five patients no abnormality could be detected. We had five conversions, two due to hidden retrocaecal appendix, two due to gangrenous base and in one we had bleeding from appendicular artery. Most of these conversions are in our learning curve period.

For laparoscopic appendicectomy mean time taken was 44.1 min. The analgesic and antibiotic requirement was significantly low in laparoscopic appendectomy. The postoperative stay and return to normal activity was early. No patient of laparoscopic group had port site infection.

### Laparoscopic orchiopexy

Forty-three children with 51 nonpalpable undescended testes underwent laparoscopy in our center. Among this, four had absent testes. 32 of the 47 (68.1%) testes were intra-abdominal as against varying reports ranging from 38.1 to 81.8%. A high intra-abdominal testis is one, which is more than 2.5 cm from the internal ring. Twelve (37.5 %) of our 32 intra-abdominal testes were high and all these were treated by a staged Stephen–Fowler laparoscopic orchiopexy. All the patients who had undergone orchiopexies showed good vascular flow on postoperative Doppler examination. Postoperative atrophy was seen in two testes on follow up and another testis was found to be retracted into the inguinal canal for which an inguinal exploration and orchiopexy was done at 6 months postlaparoscopic orchiopexy. Both atrophic testes were seen after a staged procedure while the retraction was seen after a single stage laparascopic orchiopexy. The overall success rate of laparoscopic orchiopexy is 93.61%.

### Laparoscopic cholecystectomy [Figure 3]

**Figure 3:**
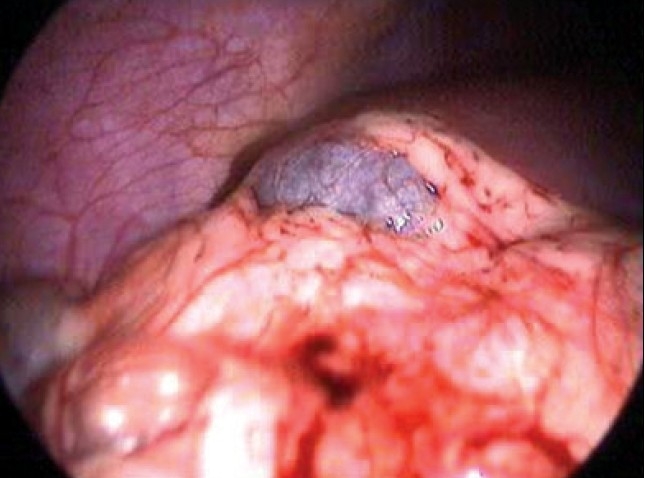
Extensive omental adhesions in cholecystitis

Since the introduction of ultrasonography, cholelithiasis in children is being increasingly recognized and referred to us. In our series 29 patients underwent laparoscopic cholecystectomy. The youngest patient in the study was a 2½-year-old boy. Ninteen patients were males and ten were females. Symptomatic gallstones were present in 26 patients. Acalculous cholecystitis was the indication for cholecystectomy in only three patients. Four patients had hereditary spherocytosis and one had sickle cell anaemia.

Operative time varied from 45 to 180 min with a mean time of 75 min and as we gained experience the operative time reduced. Of the 29 patients, 25 patients were discharged on second postoperative day. Twenty-six patients returned to their normal activity i.e., going to school and play with in 8 days. We had three conversions, two due to bleeding and in one case we had bile duct injury, which was fortunately identified and repaired during same sitting.

### Video assisted thoracoscopic surgery

In our series 109 children underwent VATS. VATS for empyema was done in 95 cases of which 15 cases were converted to open procedure due to very thick peel and badly damaged underlying lung. In cases who had presented early by the end of 5–7 days of beginning of thoracic suppurative disorder, toileting of pleural cavity was done by first clearing the pleural cavity of collection and then removing all adhesions between the lung and the parietal pleura. Suction irrigation cannula itself was used to remove the tough as well as the fibrinous adhesions. In case where the patients had presented later than seven days, the pleural peel appeared to be thick and densely adherent to the underlying lung as well as to the thoracic wall and diaphragm and these may require decortication.

There were 59 male patients and 36 female patients. The average age of the patients was 6 years with a range from 11 months to 12 years. CT scan was done for all the patients preoperatively. CT scan documented the disease process in a better way and facilitated in placement of trocars.

**Figure 4:**
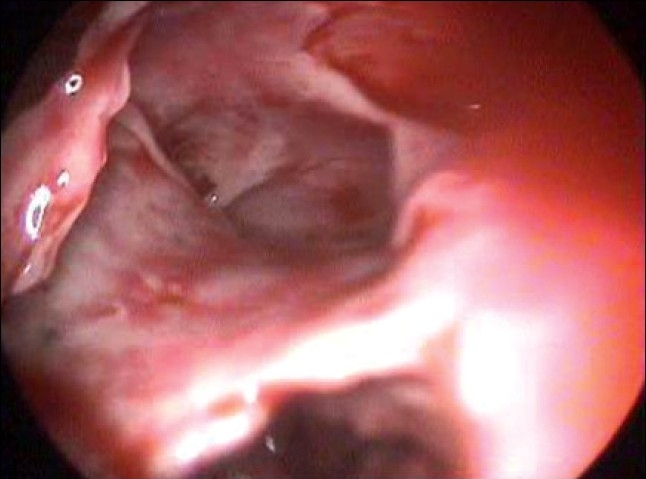
Pleural peel in empyema

We had 44 patients of empyema where we did Primary VATS (patients in whom tube thoracostomy was not done preoperatively). Of these only four patients required a conversion to thoracotomy. Fifty-one patients had been initially treated with a tube thoracostomy and out of these 11 were converted to thoracotomy. These patients had collapsed lungs and extremely dense fibrous peel [Figure 4]. These findings suggest that an initial tube thoracostomy increases the chances of conversion. Chances of conversion are also more if the disease process is more than 7 days and increases as the duration increases. Total conversion rate in our series was 15.7%. The mean operating time was 90 min (ranging between 150 and 45 min). In all patients intercostal drainage tubes were kept postoperatively. The average time that the intercostal tube was kept was for 3.5 days.

Good lung expansion in the immediate postoperative period was seen in 92% of cases. Two patients of VATS had postoperative residual collection for which Re-do VATS was done after which there was complete expansion. Three patients developed pneumothorax 6 weeks, 8 weeks and 4 months after VATS, which was treated with simple tube drainage. All patients have been clinically asymptomatic since their discharge and have been followed up on an average of 12–36 months. The chest X-ray of all patients is normal. There was no mortality in our study. The average duration of hospital stay has been 8 days postoperatively in empyema.

### VATS in lung abscess

In our series we had four cases of lung abscess not responding to parenteral antibiotics (>2 weeks) in which VATS aspiration was done. All the patients responded very well to antibiotics after aspiration.

### VATS lung biopsy

**Figure 5:**
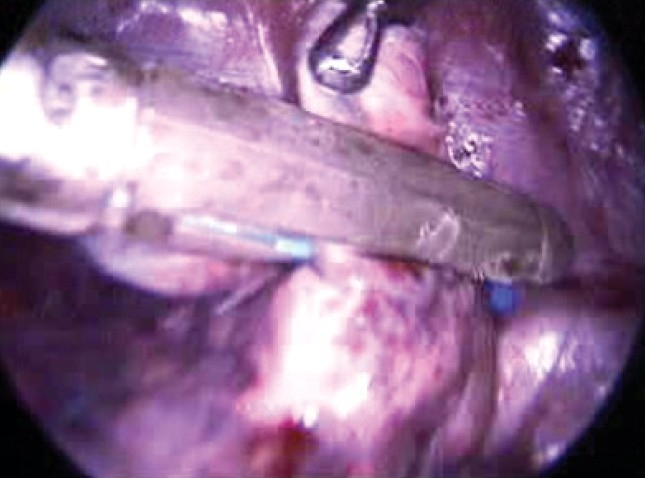
Endoscopic stapler for lung biopsy

We have done VATS lung biopsy [Figure 5] in five cases. In two cases endoscopic stapler was used and in three vicryl loop was used. We find it to be very good procedure with least morbidity. Histopathology revealed Tuberculosis in three cases, sarcoidosis in one case and interstitial lung disease in one case.

### VATS in pulmonary hydatid

We have treated three children with primary pulmonary hydatid cysts (total of six cysts). For four cysts we did VATS assisted minithoracotomy with enucleation and capittonage and two with VATS assisted limited resection.

### VATS in trauma

We had a 6-year-old boy who presented with blunt injury to chest. Chest X-ray revealed Right pneumothorax. Inspite of 3 days of intercostal drainage there was no improvement. On VATS, we found the tip of endotracheal suction tube introduced by the anaesthetist in pleural cavity. Thoracotomy revealed complete transection of right main bronchus, which was repaired. The child had uneventful recovery and is doing well.

### Neonatal ovarian cysts

**Figure 6:**
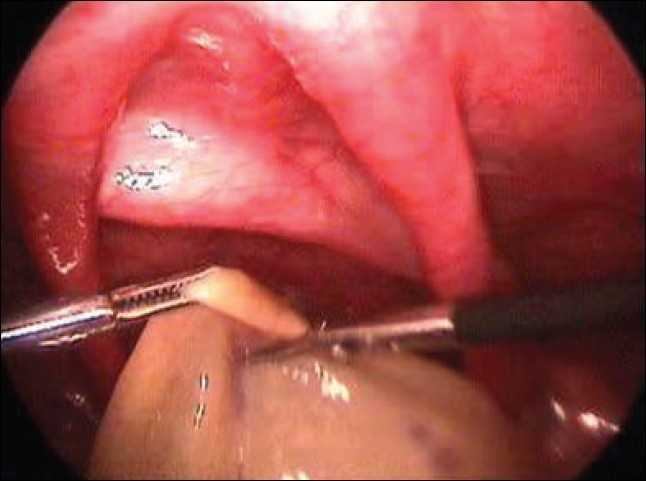
Deroofing of neonatal ovarian cyst with 2 mm instruments

We encountered three cases of neonatal ovarian cysts, which were more than 5 cm in size. Two cases underwent laparoscopic aspiration and deroofing and in other case laparoscopic oophorectomy was done because of torsion [Figure 6]. Histopathologically, all the three cysts turned out to be Follicular cyst. There is no recurrence of the cyst with a mean follow up of 15 months till now.

### Lap assisted pull through

Ninteen cases underwent Lap assisted pull through of which 13 cases were Hirschsprungs and six were High anorectal malformations. Three cases were converted to open procedure.

### Laparoscopy in solid organs

In present series, six patients underwent nephrectomy, five underwent splenectomy and one case adrenalectomy.

### Laparoscopic mesenteric lymph node biopsy

We have done laparoscopic mesenteric lymph node biopsy in eight cases. In five cases we delivered the mesentery through the umbilical port and did extracorporeal biopsy and in three cases we took biopsy within the abdomen. We have found laparoscopy to be a very good diagnostic tool, which could yield adequate tissue for histopathological confirmation with least morbidity.

## DISCUSSION

The horizon of Paediatric laparoscopic surgery has widened so much that most of the intra-abdominal pathologies in children can now be safely tackled laparoscopically. The different indications for paediatric laparoscopy can be divided into four groups depending upon the benefit of laparoscopy compared to open surgery.

Group I: Operations where the laparoscopic approach provides an undoubted benefit and has replaced open intervention like Cholecystectomy, Cryptorchidism (diagnostic and therapeutic), Appendicectomy, Fundoplication, Adrenelectomy, Varicocele operations and Nephrectomy for benign conditions.

Group II: Laparoscopy appears beneficial and safer but more information is warranted. These include Hernia repair, Adhesiolysis, Treatment of V.P. Shunt complications and Pyeloplasty.

Group III: Operations, which are currently under evaluation and should not be attempted outside clinical trials like TEF repairs, Ureteric implantations, Intestinal resection and anastomosis.

Group IV: Unsuitable by laparoscopy like Major cancer resections and Hepatic resections. These are the operations where access trauma forms only a small percentage of total operative insult.

### Inguinal hernia repair

Inguinal hernia repair is one of the most common operations performed in children [Table 2]. Laparoscopic inguinal hernia repair in children is often considered controversial but as the surgeon gains experience it has been proved to be feasible, safe and reliable technique. A contralateral PPV is present in a significant number of children and laparoscopy offers safe alternative to treat both sides at the same sitting.[1,2] An internal opening of less than 2 mm can be left alone as it is unlikely to cause a hernia.[4]

**Table 2:** Comparative study with similar series

	Schier et al.[4]	Montupet and Esposito[3]	Current

No. of repairs	223	339	259
Age	3 weeks to 13 years	8 months to 14 years	1.5 months to 12 years
Median age	2.0 years	4.2 years	4.6 years
No. of girls	58 (26%)	5 (1%)	56 (21.6%)
No. of boys	185 (74%)	334 (99%)	201 (77.6%)
Contra-lateral patent vaginalis	Rt. side hernia – Lt CPPV = 147 B-137 G-10 Lt. side hernia – Rt. CPPV = 152 B-131 G-21	Overall 60 out of 339	Rt. side hernia – Lt CPPV = 32 Lt. side hernia – Rt. CPPV =21
Direct hernia	9 (4%)	4 (1.2%)	3 (1.15%)
No hernia	1.9%		4 (1.54%)
Suture used	4-0 Nonabsorbable	4-0 Nonabsorbable	3-0 Nonabsorbable
Operating time (unilateral/bilateral)	14/21 min	13/23 min	25/35 min
Recurrence	8 (3.5%)	12 (3.5%)	9 (3.5%)
Hydrocele	9 (4%)	4 (1.2%)	4 (1.54%)
Testicular atrophy	1 (0.5%)	–	–

### Appendicectomy

Since MacKernan and Saye did first laparoscopic appendicectomy in 1988, it has rapidly gained acceptance and has shown to be safe and effective technique. If only the size of the scar is compared, laparoscopy seems to have very few advantages over classic Mc Burney's incision. However, there is very little doubt that a laparoscopic appendicectomy has reduced postoperative morbidity and early return to school with virtually scarless abdomen and has a significantly low incidence of postoperative adhesions. [5–7]

Sometimes in the presence of acute or chronic recurrent abdominal pain syndrome, laparoscopy shows a normal appendix. If another disease, which explains the pain syndrome is found and treated, the appendix can be left alone. On the other hand, if no anomaly is found it seems more judicious to remove the appendix. The argument in favour of this approach is endoappendicitis, which reaches only the mucosa. Most of the ‘normal-looking appendix’, which were removed showed either fecoliths or worms or histopathological evidence of chronic inflammation.

### Orchiopexy

Laparoscopy has been accepted as the surgical procedure of choice in children with nonpalpable undescended testis. This has proved to be a safe and reliable diagnostic test for nonpalpable undescended testis with an accuracy of 100%, which no radiological method could achieve. Since 25–56% of nonpalpable testes are reported to be vanishing, laparoscopy avoids extensive retroperitoneal dissection in these patients. The other important advantage is the decision about the technique of orchiopexy in intra-abdominal testis where a single or double staged laparoscopic procedure is done depending on whether the testis is high or low lying within the abdomen. [8–11]

### Cholecystectomy

In our series nonhaemolytic cholelithiasis is the commonest indication for cholecystectomy in children. Even other studies show nonhaemolytic cholelithiasis as the commonest indication for cholecystectomy.[12] By comparison with conventional open cholecystectomy, laparoscopic approach appears to be associated with minimal morbidity, shorter hospital stay, earlier return to school and a better cosmetic result [Table 3].[13]

**Table 3:** Showing comparison of parameters between present study and other studies

Variable	Kim et al.[12]	Jawad et al.[13]	Present study
1. Parenteral analgesia (days) LC	1.8 ± 1.4		1
OC	2.7 ± 0.9		3
2. Operating time (minutes) (Mean) LC	129.8 ± 74.1	89.81 ± 21.89	75
OC	86.4 ± 30.9		50
3. Duration of stay (days) (Mean) LC	2.0±1.8	1.68±0.46	3.35
OC	6.32.9	6.07±0.30	8.5
4. Resume activity at LC			8
(Days) OC			21
5. Wound infection LC			0
(Number) OC			1

Abbreviations: OC, Open Cholecystectomy; LC, Laparoscopic Cholecystectomy.

Unpaired *t*-test applied for operating time and duration of stay.

For operating time: *t*-value is 2.131 and *P*-value is 0.0427 (significant).

For duration of stay: *t*-value is 4.286 and *P*-value is 0.0002 (significant).

### VATS

In empyema cases, it was initially thought that proper decortication and debridement can only be accomplished by open thoracotomy and therefore earlier recommendations were for early thoracotomy and decortication in all cases of empyema in children. Unfortunately, these procedures are associated with significant morbidity and mortality and prolonged hospitalization. VATS has recently regained popularity as an attempt to decrease the morbidity associated with open decortication. [14–16]

VATS lung biopsy is more efficient as it saves operative time and provides good visualization of lung and pleural surfaces in addition to reducing postoperative pain and hospital stay. Fiona et al. concluded that sufficient tissue could be provided for both microbiological and histological study.[17]

VATS assisted minithoracotomy is being described as an alternative in the management of pulmonary hydatid. The postoperative morbidity is much reduced compared to the classical thoracotomy in the surgical treatment of pulmonary hydatid cysts in children.[18]

### Neonatal ovarian cyst

Small simple ovarian cysts fewer than 5 cm in diameter can be observed carefully with serial ultrasonography. But, all symptomatic and complex ovarian cysts and simple cysts over 5 cm in diameter in addition to smaller cysts less than 5 cm showing no decrease in size should be considered as a surgical indication to rescue the ovarian tissue. Laparoscopy is well tolerated by newborns. In addition to confirming the diagnosis, laparoscopy can be used for aspiration, deroofing, cystectomy and oopherectomy in cases of ovarian cyst.[19,20]

## CONCLUSION

MAS is here to stay. This is as truer in Paediatric surgery as in adults. It is needless to mention the advantages of MAS. With proper judgment and self-assessment, an exciting and stimulating time exists in the future for surgeons incorporating MAS into their clinical practice. Nevertheless conversion to open procedure in case of difficulty, anomalous anatomy or unsuspected pathology by killing the personal ego represents sound surgical judgement and is a testimony to a surgeon's conservative and more importantly ‘safe approach’.
